# Diastolic dysfunction is more apparent in STZ-induced diabetic female mice, despite less pronounced hyperglycemia

**DOI:** 10.1038/s41598-018-20703-8

**Published:** 2018-02-05

**Authors:** Chanchal Chandramouli, Melissa E. Reichelt, Claire L. Curl, Upasna Varma, Laura A. Bienvenu, Parisa Koutsifeli, Antonia J. A. Raaijmakers, Miles J. De Blasio, Cheng Xue Qin, Alicia J. Jenkins, Rebecca H. Ritchie, Kimberley M. Mellor, Lea M. D. Delbridge

**Affiliations:** 1 Department of Physiology, University of Melbourne, Melbourne, Victoria Australia; 2 School of Biomedical Sciences, University of Queensland, Brisbane, Queensland Australia; 3 Heart Failure Pharmacology, Baker Heart & Diabetes Institute, Melbourne, Victoria Australia; 4 Department of Physiology, University of Auckland, Auckland, New Zealand; 5 NHMRC Clinical Trials Centre, University of Sydney, Sydney, New South Wales Australia; 6 School of Biosciences, University of Melbourne, Melbourne, Victoria Australia; 7 Department of Pharmacology and Therapeutics, University of Melbourne, Melbourne, Victoria Australia; 8 National Heart Centre, Singapore, Singapore

## Abstract

Diabetic cardiomyopathy is a distinct pathology characterized by early emergence of diastolic dysfunction. Increased cardiovascular risk associated with diabetes is more marked for women, but an understanding of the role of diastolic dysfunction in female susceptibility to diabetic cardiomyopathy is lacking. To investigate the sex-specific relationship between systemic diabetic status and *in vivo* occurrence of diastolic dysfunction, diabetes was induced in male and female mice by streptozotocin (5x daily i.p. 55 mg/kg). Echocardiography was performed at 7 weeks post-diabetes induction, cardiac collagen content assessed by picrosirius red staining, and gene expression measured using qPCR. The extent of diabetes-associated hyperglycemia was more marked in males than females (males: 25.8 ± 1.2 vs 9.1 ± 0.4 mM; females: 13.5 ± 1.5 vs 8.4 ± 0.4 mM, p < 0.05) yet *in vivo* diastolic dysfunction was evident in female (E/E′ 54% increase, p < 0.05) but not male diabetic mice. Cardiac structural abnormalities (left ventricular wall thinning, collagen deposition) were similar in male and female diabetic mice. Female-specific gene expression changes in glucose metabolic and autophagy-related genes were evident. This study demonstrates that STZ-induced diabetic female mice exhibit a heightened susceptibility to diastolic dysfunction, despite exhibiting a lower extent of hyperglycemia than male mice. These findings highlight the importance of early echocardiographic screening of asymptomatic prediabetic at-risk patients.

## Introduction

Diabetes is a condition of epidemic proportions, with cardiovascular mortality the leading cause of death in diabetic patients. Recognition of diabetic cardiomyopathy as a primary cardiac pathology is now well established, where cardiac functional and structural abnormalities are evident independent of coronary artery disease and hypertension^1–4^. Population-based studies have identified that diastolic dysfunction is prevalent in more than 50% of asymptomatic diabetic patients^5–7^, and linked to an increased risk of heart failure and mortality in diabetes, independent of systolic functional decline^8^. Thus, while it is clear that diastolic dysfunction is an early manifestation of diabetic cardiomyopathy and prognostic of later adverse outcomes, current clinical guidelines do not support routine echocardiographic screening of diastolic function in asymptomatic diabetic patients^9^.

Clinical evidence suggests that diabetic women exhibit a higher susceptibility to cardiovascular morbidity and mortality than diabetic men. The Framingham Heart Study first reported a higher relative incidence of heart failure in diabetic women compared to men (5.2-fold vs 2.2-fold increased incidence relative to non-diabetic for women and men, respectively)^10^. Subsequent studies have shown that diabetes increases the risk of cardiovascular events to a greater extent in women than in men^11–14^. Even in diabetic patients with comparable glycemic control, an increased cardiovascular risk factor profile is reported in diabetic females relative to males^15^. Heightened cardiovascular risk in females does not appear to be dependent on vascular pathology—in adolescent type 1 diabetic patients with no evidence of hypertension or coronary artery disease, cardiac structural remodeling and diastolic dysfunction was evident in females but not male subjects^16^. Diagnosis of diabetes and subsequent evaluation of cardiac risk may be delayed in females, as elevated fasting glucose is more common in diabetic males^17,18^. Glucose dysregulation in females is more frequently characterized by impaired glucose tolerance^18–20^. Interestingly, impaired glucose tolerance has been shown to be a stronger predictor of left ventricular diastolic dysfunction than abnormal fasting glucose^7^.

The available experimental evidence for sex differences in cardiac dysfunction in diabetes is somewhat consistent with the clinical epidemiological data. The only study to date to directly investigate sex differences in diastolic dysfunction *in vivo* in an experimental animal model of diabetes demonstrated that streptozotocin-induced diabetic female mice exhibited a more marked decrease in the echocardiographic E/A ratio parameter relative to males, in a setting where blood glucose levels were matched between sexes^21^. Other studies have confirmed the presence of diastolic dysfunction in female diabetic rodents, but no male group was included for comparison in these studies^22,23^. Differential structural and molecular abnormalities are evident in male and female obese type 2 diabetic mice (*db/db*), with earlier onset of cardiomyocyte hypertrophy despite no change in hypertrophic gene expression (β-myosin heavy chain, brain natriuretic peptide) in females^24^. Interestingly, evaluation of hearts or cardiomyocytes isolated from diabetic rodents have failed to recapitulate exacerbated diabetic cardiac pathology in females relative to males^25–28^, highlighting the importance of neurohumoral involvement in sex differences observed *in vivo*.

Given the clinical evidence that cardiovascular morbidity and mortality is higher in diabetic women, and diastolic dysfunction is an important prognostic indicator of mortality in diabetes, we sought to investigate the sex specific relationship between systemic indicators of diabetic status and *in vivo* occurrence of diastolic dysfunction and related cardiac structural pathology in male and female mice. This study provides the first evidence that exacerbated diastolic functional pathology is not indexed by systemic glycemic disturbance in STZ-induced diabetic female mice.

## Results

### Diabetes-induced glycemic dysregulation is less pronounced in female mice

**Figure 1:**
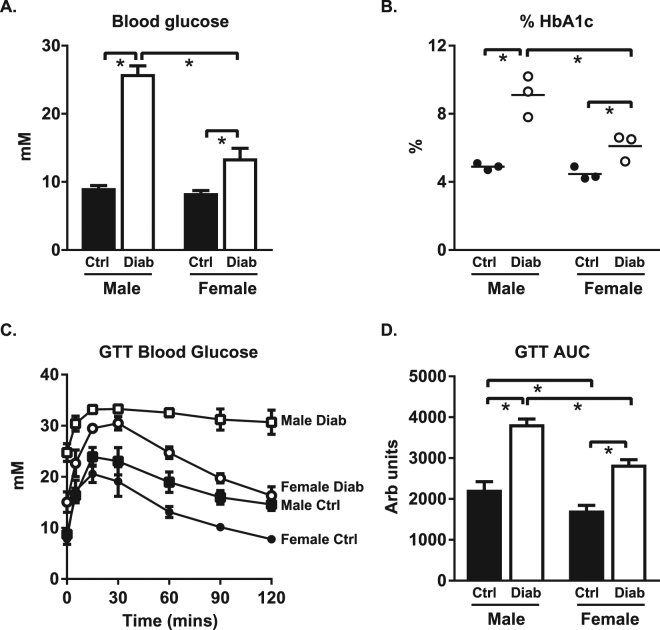
Hyperglycemia and impaired glucose tolerance are less pronounced in diabetic females than males. (**A**) Blood glucose levels (n = 14–17/group). (**B**) Glycated hemoglobin levels (%, n = 3/group). (**C**) Blood glucose disappearance following 1.5 g/kg glucose load (n = 5–6/group). Note, in some instances error bars are not discernable as they fall within symbol shapes. (**D**) Area under the glucose tolerance curve (n = 5–6/group). Data are presented as mean ± SEM. *p < 0.05, 2-way ANOVA, annotated with LSD *post hoc* analyses.

To characterize sex differences in systemic responses to diabetes, glycemic status was examined in STZ-induced diabetic male and female mice, at 8 weeks post-STZ injections. The extent of diabetes-induced increase in blood glucose levels was lower in females than in males (diabetic females: 1.6 fold control vs. diabetic males: 2.8 fold control, p < 0.05, Fig. 1A). Similarly, the extent of diabetes-induced increase in % HbA1c was less pronounced in females than in males (diabetic females: 1.4 fold control vs. diabetic males: 1.9 fold control, p < 0.05, Fig. 1B). Glucose tolerance tests also confirmed delayed glucose clearance with diabetes, an effect which was less marked in females than in males (p < 0.05, Fig. 1C,D).

### Cardiac diastolic and systolic dysfunction is evident in diabetic female but not male mice

**Figure 2:**
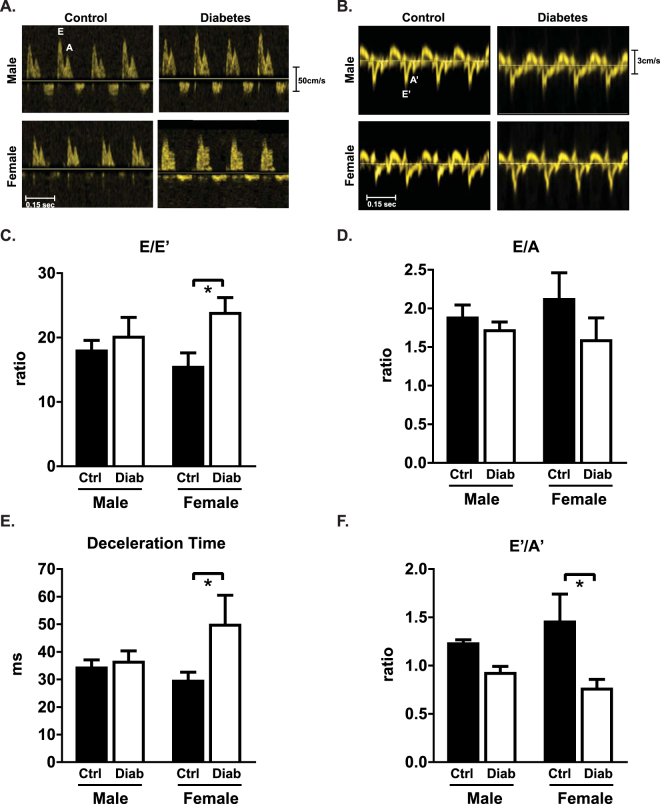
Diastolic dysfunction is evident in diabetic female but not male mice. (**A**) Representative echocardiography traces from pulse-wave (blood flow) Doppler imaging. (**B**) Representative echocardiography traces from A4C view of mitral valve tissue Doppler. (**C**) Ratio of flow Doppler E wave amplitude to tissue Doppler E′ wave amplitude. (**D**) Ratio of flow Doppler E wave to A wave amplitude. (**E**) Mitral valve flow Doppler deceleration time. (**F**) Ratio of tissue Doppler E′ wave to A′ wave amplitude. Data are presented as mean ± SEM. n = 5/group. *p < 0.05, 2-way ANOVA, annotated with LSD *post hoc* analyses.

To determine whether sex disparities in the systemic response to diabetes resulted in differential cardiac diastolic functional outcomes, *in vivo* flow and tissue Doppler imaging was performed in male and female diabetic mice at 7 weeks following STZ injections. Representative traces of flow Doppler and tissue Doppler velocities are shown in Fig. 2A,B respectively. Diabetes induced an increase in the ratio of early transmitral valve flow velocity (E wave) to early mitral annulus tissue velocity (E′ wave) in female but not male mice, an important clinical index of diastolic dysfunction (E/E′, diabetic females: 54% increase, p < 0.05, Fig. 2C). Changes in the mitral valve early to late filling velocity E/A ratio did not reach significance (Fig. 2D). Diabetes induced a female-specific prolongation of E-wave deceleration time (Dec T, diabetic females: 68% increase, p < 0.05, Fig. 2E). The ratio of early to late diastolic mitral annulus tissue velocity was significantly lower with diabetes in female but not male mice (E′/A′, diabetic females: 47% decrease, p < 0.05, Fig. 2F). Collectively, these data suggest that diastolic dysfunction is more apparent in STZ-induced diabetic females than males, despite less pronounced hyperglycemia. Additionally, it should be noted that all Doppler records contained evidence of a third ‘negative deviation’ wave form prior to the S’. The occurrence of this wave has been noted in larger animals and human subjects^29,30^, and whilst observable in the small number of murine studies where Doppler images are presented (for example^31^), it has not been previously commented or analysed.

**Figure 3:**
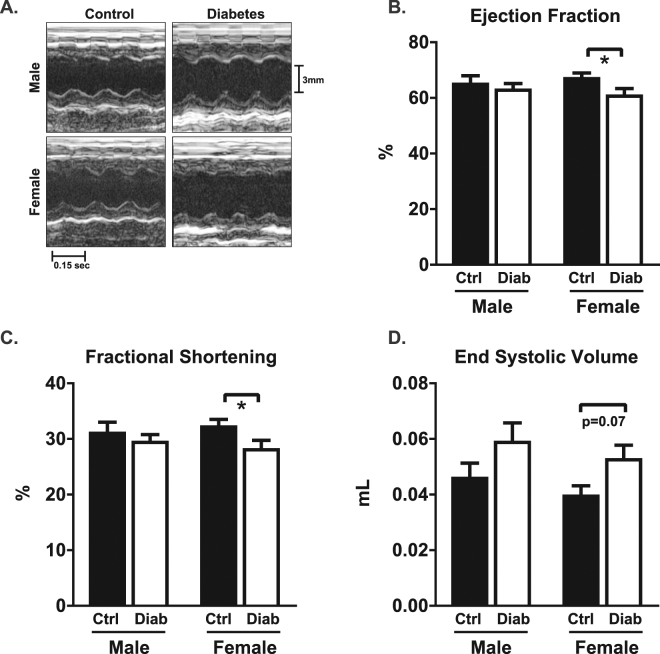
Mild systolic dysfunction is evident in diabetic female but not male mice. (**A**) Representative M-mode traces from left ventricular short axis view. (**B**) Ejection fraction. (**C**) Fractional shortening. (**D**) End systolic volume. Data are presented as mean ± SEM. n = 12 15/group. *p < 0.05, 2-way ANOVA, annotated with LSD *post hoc* analyses.

*In vivo* systolic function was assessed from M-mode parasternal short axis echocardiography. Representative traces are shown in Fig. 3A. Systolic function was preserved in male diabetic mice. A modest diabetes-induced reduction in ejection fraction (9% decrease) and fractional shortening (13% decrease) was evident in females (p < 0.05, Fig. 3B,C). A trend for diabetes-induced increased end systolic volume in females was evident (ESV, diabetic females: 33% increase, p = 0.07, Fig. 3D). These data suggest that mild systolic dysfunction is evident in female STZ-induced diabetic mice, but cardiac dysfunction is yet to emerge at this 7 week diabetic time point in males.

### Diabetes-induced cardiac structural remodeling is similar in males and females

**Figure 4:**
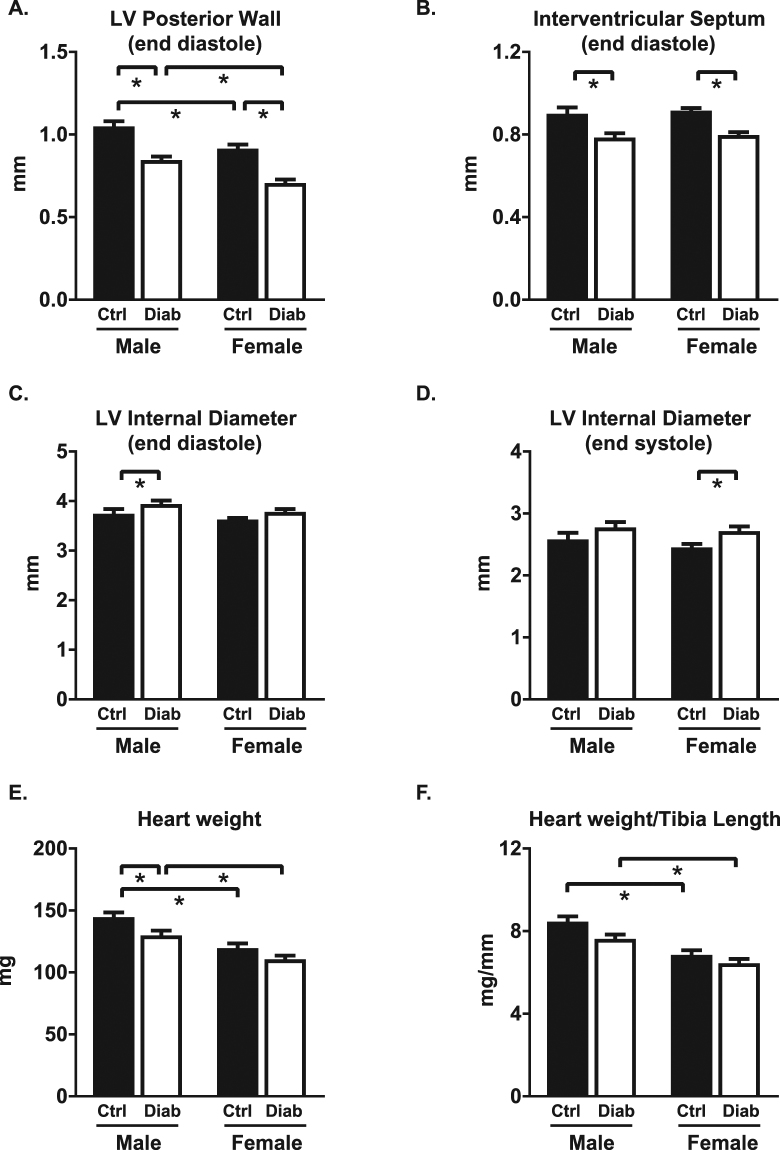
Cardiac morphological responses to diabetes are similar in male and female mice. *In vivo* echocardiography assessment of (**A**) left ventricular posterior wall thickness at end diastole, (**B**) interventricular septal wall thickness at end diastole, (**C**) left ventricular internal diameter at end diastole, (**D**) left ventricular internal diameter at end systole. (**E**) Dissected heart weight. (**F**) Heart weight (mg) normalized to tibia length (mm). Data are presented as mean ± SEM. n = 12–16/group. *p < 0.05, 2-way ANOVA, annotated with LSD *post hoc* analyses.

Anatomic changes in heart geometry were examined by measuring septal and posterior wall thickness from M-mode echocardiography and cardiac weight indices. Left ventricular posterior wall thickness at end diastole was reduced with STZ-induced diabetes (diabetic males: 19% decrease; diabetic females: 23% decrease; p < 0.05, Fig. 4A) and a similar differential was observed in posterior wall thickness at end systole (Table 1). Interventricular septal wall thickness was reduced with diabetes at end diastole and at end systole to a similar extent in both males and females (11–13% decrease, p < 0.05, Fig. 4B and Table 1). A modest but significant increase in left ventricular internal diameter was evident in diabetic males but not females at end diastole (5% increase, p < 0.05, Fig. 4C), and in diabetic females but not males at end systole (11% increase, p < 0.05, Fig. 4D). As expected, heart weight, ventricle weight (combined right and left ventricle) and heart weight normalized to tibia length were smaller in females than males (p < 0.05, Fig. 4E,F and Table 1). Heart weights normalized to body weight were not different (Table 1). A significant diabetes-induced decrease in heart weight and ventricle weight was detected in males but not females (p < 0.05, Fig. 4E, Table 1). Collectively these findings suggest that STZ-induced diabetes was associated with similar wall thinning and chamber dilation in male and female mice, even in the context of marked differences in systemic glycemic status.

**Table 1:** *In vivo* echocardiography cardiac functional and structural characteristics in diabetic male & female mice.

	Male		Female	
	Ctrl	Diabetes	Ctrl	Diabetes
E wave (mm/s)	504 ± 39	412 ± 39	454 ± 55	521 ± 82
A wave (mm/s)	278 ± 36	245 ± 28	226 ± 30	352 ± 72
E′ wave (mm/s)	29.0 ± 2.2	21.8 ± 2.5*	28.7 ± 1.3	21.7 ± 2.0*
A′ wave (mm/s)	23.4 ± 2.0	23.1 ± 1.5	22.6 ± 4.2	30.2 ± 6.3
End diastolic vol. (mL)	134 ± 8.8	154 ± 8.0*	121 ± 3.5	137 ± 5.8
Heart rate (bpm)	478 ± 23	437 ± 11	486 ± 15	460 ± 18
Stroke vol. (mL)	87.3 ± 6.6	96.7 ± 4.1	80.7 ± 2.2	84.6 ± 3.3^#^
Cardiac output (mL/s)	0.71 ± 0.05	0.71 ± 0.03	0.66 ± 0.02	0.65 ± 0.05
IVSs (mm)	1.32 ± 0.04	1.17 ± 0.03*	1.40 ± 0.03^#^	1.22 ± 0.04*
LVPWs (mm)	1.30 ± 0.04	1.10 ± 0.04*	1.17 ± 0.03^#^	0.91 ± 0.05*^#^
Relative wall thickness (ratio)	0.53 ± 0.02	0.42 ± 0.01*	0.51 ± 0.01	0.40 ± 0.01*
Ventricular weight (mg)	130 ± 4.0	115 ± 3.2*	111 ± 4.2^#^	95.9 ± 2.9*^#^
Heart weight/body weight (mg/g)	4.72 ± 0.1	4.71 ± 0.1	4.99 ± 0.1	4.87 ± 0.1

Mitral valve flow velocity, early ventricular filling phase (E wave) and during atrial contraction (A wave); mitral valve tissue movement velocity, early ventricular filling phase (E′ wave) and during atrial contraction (A′ wave), interventricular septal wall thickness at end systole (IVSs), left ventricular posterior wall thickness at end systole (LVPWs), relative wall thickness (RWT). Dissected ventricular weight (left and right ventricle combined) and heart weight normalized to body weight. Data are presented as mean ± sem. n = 5/group (Doppler parameters), n = 12–16/group (M-mode parameters). *p < 0.05 *vs* Ctrl, ^#^p < 0.05 *vs* male, 2-way ANOVA, annotated with LSD *post hoc* analyses.

**Figure 5:**
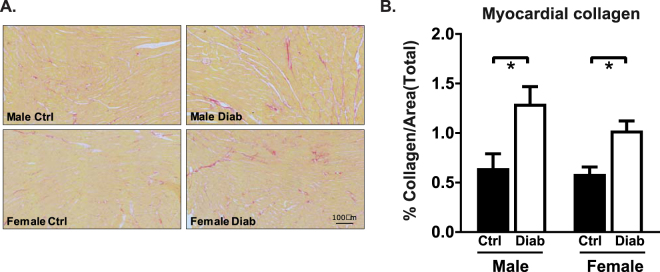
Cardiac fibrotic infiltration is similar in diabetic male and female mice. (**A**) Representative picrosirius red-stained transverse left ventricular sections. (**B**) Total collagen content (as a % of image area) calculated from picrosirius red-stained sections of ventricular tissue (n = 5 heart/group). Data are presented as mean ± SEM. *p < 0.05, 2-way ANOVA, annotated with LSD *post hoc* analyses.

To determine whether the accentuated diabetic female functional pathology corresponded to more marked fibrotic structural remodeling, myocardial collagen content was assessed 8 weeks post-STZ injections. Representative images of picrosirius red stained images from transverse sections of the left ventricle are shown in Fig. 5A. Diabetes-induced fibrosis was similar in male and female mouse heart sections (% collagen, diabetic males: 2.0 fold control, diabetic females: 1.7 fold control, p < 0.05, Fig. 5B). Thus accentuated diabetes-associated cardiac dysfunction in females cannot be attributed to exacerbated myocardial fibrosis.

### Sex differences in expression of cardiac glucose handling and autophagy genes in diabetic mice

**Figure 6:**
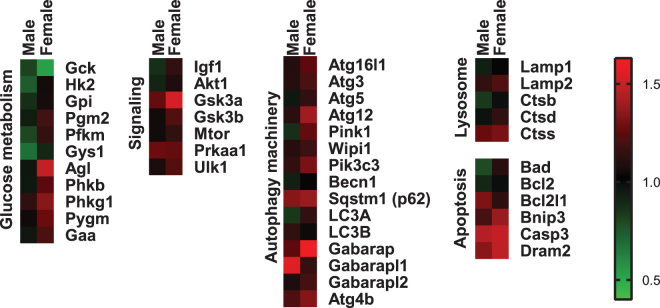
Differential cardiac gene expression in diabetic male and female mice. RT profiler data from 44 genes in heart tissue from male and female diabetic mice. Fold change diabetes vs control for male and female groups depicted by graded red (upregulation) and green (downregulation), n = 3/group.

**Figure 7:**
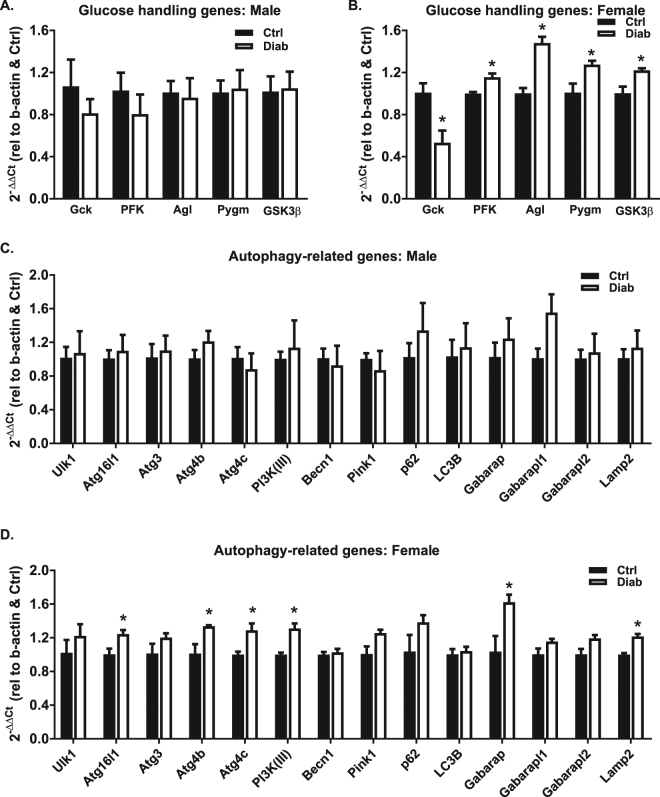
Differential cardiac glucose handling and autophagy gene expression in diabetic male and female mice. Glucose metabolic mRNA expression in male (**A**) and female (**B**) diabetic mouse hearts. Autophagy-related mRNA expression in male (**C**) and female (**D**) diabetic mouse hearts. Data are presented as mean ± SEM. n = 3/group. *p < 0.05, Students T-test.

To explore whether changes in cardiac gene expression profiles may underlie female susceptibility to cardiac dysfunction in diabetes, RT profiler qPCR arrays were performed on male and female STZ-induced diabetic mouse heart cDNA. A heatmap depicting the fold change in mRNA expression of diabetes vs control is presented in Fig. 6 (red: upregulation, green: downregulation). Key genes involved in glucose handling and autophagy with statistically significant diabetes-induced differences for male or female mice are presented in Fig. 7. Female mice exhibited diabetes-induced downregulation of *glucokinase* (Gck, 47% decrease, p < 0.05) and upregulation of *phosphofructokinase 2* (PFK, 16% increase, p < 0.05), *glycogen debranching enzyme* (Agl, 48% increase, p < 0.05), *glycogen phosphorylase* (Pygm, 26% increase, p < 0.05) and *glycogen synthase kinase 3β* (GSK3β, 22% increase, p < 0.05) (Fig. 7B). No significant differences in glucose handling or autophagy-related gene expression were observed in male diabetic mouse hearts (Fig. 7A,C). In females, diabetes induced significant upregulation of multiple autophagy machinery genes (*Atg16l1, Atg4c, Atg4b, PI3K(III), Gabarap, Lamp2*, Fig. 7D), indicative of an increased drive for autophagy processes. These data provide preliminary evidence that heightened diastolic dysfunction in STZ-induced diabetic female mice is linked with female-only upregulation of key genes involved in glucose metabolism and autophagy which may provide a molecular basis for female-specific cardiac dysfunction observed in this study.

## Discussion

This study provides the first experimental demonstration that females are more susceptible to diastolic dysfunction in diabetes, despite a lower extent of hyperglycemia. These findings recapitulate the clinical observations that diabetic females exhibit heightened cardiac dysfunction and provide new insights into the underlying mechanisms involved. At this 7–8 week time-point of STZ-induced diabetes progression, male mice exhibited marked hyperglycemia yet appeared to be relatively protected from cardiac dysfunction. In contrast, STZ-induced diabetic female mice exhibited severe diastolic dysfunction and mild systolic dysfunction, despite a less pronounced glycemic insult. Cardiac morphological remodeling in STZ-induced diabetes was similar between males and females. Female-specific upregulation of cardiac glucose handling and autophagy genes was evident, which may provide a molecular basis for female cardiac vulnerability in this setting. These findings contradict the conventional view that diabetic cardiomyopathy is directly related to glycemic control and highlight the importance of clinical evaluation of cardiac function in female diabetic patients with mild hyperglycemia.

Despite the clinical evidence for increased cardiovascular mortality and cardiac pathology in diabetic women^10,15,16,32^, sex-specific cardiac functional differences in experimental diabetic models have received minimal focus. Clinical studies have reported that diastolic dysfunction, more than systolic dysfunction, is predictive of heart failure and increased mortality, independent of hypertension and coronary artery disease^8^. Remarkably, with every 1 unit increase in E/E′, the hazard ratio for heart failure increases by 3%^8^. The separate concepts of female- and diabetes-related vulnerability to diastolic dysfunction are well recognized, particularly in the context of hypertension^33^, post-surgery^34^, and the ‘heart failure with preserved ejection fraction (HFpEF)’ phenomenon of increasing incidence of patients with heart failure symptoms despite normal systolic function. Yet a thorough experimental analysis of sex differences in diabetes-induced cardiac dysfunction linked with glycemic status has not been previously reported. In the present study, a heightened susceptibility to diastolic dysfunction was evident in female STZ-induced diabetic mice relative to males, even though hyperglycemia was less pronounced. These experimental findings are consistent with clinical observations that impaired fasting glucose is not a strong predictor of diastolic dysfunction^7^. Interestingly, in a diabetic experimental context where the extent of hyperglycemia was matched between male and female diabetic mice, accelerated cardiac pathology has also been observed in females linked with some indication of impaired function^21^.

In the present study, the glycemic response to streptozotocin was markedly lower in females relative to males. This finding is unlikely to be explained by a sex-specific pancreatic effect of streptozotocin, as previous studies have demonstrated that lower hyperglycemia in female mice is evident despite similar insulinitis and β-cell necrosis in C57Bl/6J mice^35^. Inconsistent findings relating to sex differences in the systemic response to streptozotocin in mice have been reported in the literature, with reports of higher^36^, lower^37^ or equivalent^21^ extent of hyperglycemia in streptozotocin-induced diabetic female relative to male mice. These discrepancies may be due to variation in dose (40–200 mg/kg, single vs multiple injections), administration route (intraperitoneal vs. intravenous), strain (ICR, C57Bl/6, CD1), or age at streptozotocin administration^21,36,37^. The present study used 5 × 55 mg/kg i.v streptozotocin injections in C57Bl/6J mice at 15 weeks of age. Impaired glucose tolerance was evident in these mice, thus supporting the contention that although streptozotocin is largely considered to induce type 1 diabetes, multiple relatively low-dose streptozotocin injections in mice induces a model which shares some phenotypic traits characteristic of both type 1 and lean type 2 diabetes, thus may offer mechanistic insights of relevance to both settings. In particular this model may be relevant to adult late onset type 1 diabetes—an immunologic condition of rising incidence^38^.

Importantly, the systemic findings from this study are consistent with the clinical literature reporting that males exhibited significantly higher fasting blood glucose levels than females at the time of diabetes diagnosis^17^. The clinical guidelines for diagnosis of diabetes in humans are fasting blood glucose ≥7 mM, or plasma glucose ≥11.1 mM at 2 hours post 75 g oral glucose load, or HbA1c levels ≥6.5% (or 48 mmol/mol), or random plasma glucose ≥11.1 mM^39^. The relatively subtle glycemic dysregulation in diabetic females vulnerable to early cardiac pathology, may not be identifiable on the basis of current diagnostic criteria. Thus implementation of sex-specific diagnostic thresholds may be more appropriate for earlier and accurate diagnosis of diabetes in women, at a disease progression stage when cardiac impairment is identifiable and earlier cardiac-risk directed intervention may be appropriate.

Despite differential functional outcomes in male and female STZ-induced diabetic mouse hearts, structural alterations in ventricular wall thickness and chamber diameter were remarkably similar in males and females. Diabetes-induced myocardial fibrotic infiltration was evident, and not different between males and females. Similar findings have been observed in type 2 diabetic mice (*db/db*) where no sex differences in myocardial fibrotic lesions were observed^24^. In the present study, the changes in wall dimensions were accompanied by increased interstitial fibrotic infiltration in the ventricular free wall in both sexes, characteristic of diabetic patients^40,41^ and experimental diabetic animal models^42–44^. Given that increased collagen deposition in the male and female diabetic hearts was associated with smaller heart weights, it is possible that fibrosis has occurred as a ‘backfill’ response to myocyte loss, but this was not directly investigated. Although this effect appears to be similar in male and female diabetic hearts, the cardiac morphological changes corresponded to a functional deficit in female but not male mice.

The mechanisms underlying clinical observations of female cardiac vulnerability in diabetes are not well established. Given that estrogen and insulin signaling actions share common regulatory pathways for maintenance of cardiomyocyte function, involvement of sex steroids in mediating differential cardiac effects of diabetes in males and females is plausible. Some information has been gleaned from experimental studies using ovariectomy to interrogate the role of systemic sex steroids in diabetic cardiomyopathy (reviewed in^45^). But recent evidence suggests that the intracardiac estrogen-androgen system is also important in cardiac pathology^46^ and understanding the role of cardiac sex steroid involvement in female susceptibility to diastolic dysfunction is an important priority.

In the present study, sex differences in diastolic dysfunction were associated with differential metabolic gene expression responses in an experimental diabetic setting. Diastolic dysfunction in diabetic females was linked with lower expression of glucokinase and upregulation of phosphofructokinase—suggesting a disconnect between the initiation and rate-limiting steps of glycolysis. In females, gene expression of glycogen breakdown enzymes (debranching enzyme and phosphorylase) was higher in diabetic hearts which could reflect a compensatory mechanism to access intracellular glucose stores in a setting of impaired glucose uptake—although different protein expression and enzyme activity responses cannot be excluded. Upregulation of glycogen degradation pathways may have the adverse outcome of intracellular glucotoxicity, and accumulation of glucose-derived metabolites for post-translational modification. Discrepant findings relating to cardiac autophagy induction in diabetes have been reported (reviewed in^47^). In the present study, diabetes-induced upregulation of autophagy genes was only evident in females. Although autophagy protein activity or protein level were not measured in the present study, higher gene expression of autophagy genes may correspond to a heightened autophagic drive in female mice. Autophagy activation could be either beneficial (by removing dysfunctional proteins and organelles and promoting cell survival), or detrimental (leading to autosis programmed cell death)^48,49^. The mechanisms underlying autophagy-induced cell death in the heart are not well defined, and autosis is emerging as a novel cell death pathway. The findings from the present study suggest that sex differences in gene regulation of glucose handling and autophagy are linked to female vulnerability to diastolic dysfunction in diabetes, and provide new leads for future mechanistic interrogation.

## Conclusions

This is the first study to demonstrate that impaired diastolic function is evident in STZ-induced diabetic female mice, despite a lower extent of hyperglycemia. Dramatic sex differences were observed in the cardiac functional outcomes of diabetes, with male mice appearing relatively protected from cardiac dysfunction, despite marked cardiac structural abnormalities and extensive hyperglycemia. Female cardiac vulnerability in diabetes is an established clinical observation, and we now show in a tightly controlled experimental setting that increased susceptibility to diastolic dysfunction cannot be predicted by conventional diabetic diagnostic measures. Our findings suggest that expression of genes involved in glucose handling and autophagy are modulated in diabetes in a female-specific manner, which may reflect an underlying disturbance in glucose metabolism and cell survival in female diabetic hearts. Understanding the mechanisms of female susceptibility to diastolic dysfunction is an important priority, and may lead to the development of sex-specific therapies in diabetes. Our findings suggest that new modeling work is required to understand the tissue deformation properties of murine myocardium, the potential value of extracting information from multiple waveforms (including the third negative deviation wave entity), and the importance of undertaking more extensive sex-specific investigations of all Doppler parameters to further validate our work. The findings from this study suggest that cardiac risk cannot be solely predicted from systemic glycemic status in diabetic patients, and females in particular may require early echocardiographic detection and treatment of diastolic dysfunction.

## Methods

### Animals and induction of diabetes

Male and female mice (C57Bl/6 background) were maintained in the Biomedical Sciences Animal Facility at the University of Melbourne, Australia. All procedures were approved by the University of Melbourne Animal Ethics Committee and all experiments were performed in accordance with the relevant guidelines and regulations of the Australian Code of Practice for the Care and Use of Animals for Scientific Purposes (2004). At 15 weeks of age, diabetes was induced by 5 consecutive daily intraperitoneal injections of 55 mg/kg streptozotocin (STZ) in 0.1 M citrate buffer as described previously^50^. Control animals were injected with 0.1 M citrate buffer vehicle. Non-fasted blood glucose levels were determined by glucometer (ACCU-CHEK Advantage, Roche, Mannheim, Germany, upper limit of detection 33.3 mM) in 10 μl blood samples collected by tail vein venopuncture in conscious mice (n = 14–17/group).

### Echocardiography

At 7 weeks post-STZ injections, transthoracic echocardiography was performed using the GE Vivid 9 Dimension echocardiography platform with a 15 MHz i13L linear array transducer (GE Healthcare, CT, USA) as described previously^51^. In brief, mice were lightly anesthetized with tribromethanol (2.5%, 0.01 ml/g i.p). Left ventricular M-mode two-dimensional echocardiography was performed in parasternal short axis view to measure left ventricular wall and chamber dimensions (interventricular septum thickness in diastole and systole (IVSd, IVSs), left ventricular internal diameter in diastole and systole (LVIDd, LVIDs) and left ventricular posterior wall thickness in diastole and systole (LVPWd, LVPWs)), and to derive systolic function parameters (ejection fraction ((EDV − ESV)/EDV) ×100), fractional shortening ((LVEDD − LVESD)/(LVEDD) ×100)), n = 12–15/group. In a subset of animals (n = 5/group), pulse wave Doppler and tissue Doppler imaging were acquired from the apical 4 chamber views to assess diastolic function parameters (velocity of early mitral inflow (E) and late mitral inflow (atrial inflow A) and E/A ratio; early diastolic velocity of mitral annulus (E′) and late diastolic velocity of mitral annulus (atrial contraction velocity A′) and E/E′ ratio and E′/A′ ratio). Three consecutive cardiac cycles were sampled for each measurement taken, evaluating wave forms from transmitral and tissue records in temporal registration to validate waveform identification, performed in two separate blinded analyses. In larger mammals and human subjects earlier seminal studies have shown that qualitative changes in E/A wave features and potential merging at high heart rate may impair measurement consistency^52–54^. Recordings with these mice involved heart rates a little under 500 bpm—avoiding the rates of 600–700 bpm where waveform fusion is most typically manifest in murine models^55,56^.

### Glucose tolerance testing

Glucose tolerance testing was conducted at 8 weeks post-STZ injections, as previously described^57^. Mice were fasted for 6 hours (n = 5–6/group), and a baseline blood glucose measurement was obtained. A glucose bolus (1.5 mg/kg glucose) was injected intraperitoneally and blood glucose levels were measured at 5, 15, 30, 60, 90 and 120 minutes post-injection. The area under the curve was calculated using trapezoidal integration as an index of glucose disappearance from plasma^58^.

### Blood and cardiac tissue collection

At 8 weeks post-STZ injections, mice were anesthetized (sodium pentobarbital, 100 mg/kg, i.p), and a thoracotomy was performed. Heparin (100 I.U., i.p.) was injected into the inferior vena cava and a blood sample collected and stored at 4 °C for HbA1c analysis and stored at −80 °C until further analysis. Hearts were rapidly excised and arrested in cold HEPES-Krebs buffer (in mM: 146 NaCl, 4.69 KCl, 11 Glucose, 0.35 NaH_2_PO_4_, 1.05 MgSO_4_, 10 HEPES; pH 7.40, 4 °C). Hearts were weighed, atria removed and ventricle weight (combined left and right ventricles) measured. A transverse ventricular mid-section (~2 mm thick) was dissected and fixed in formalin (10%) for later histological analysis. The remaining ventricular tissue was snap-frozen in liquid N_2_ for later molecular analysis. Tibias were dissected and length measured using electronic digital calipers for heart weight normalization.

### Glycated hemoglobin (HbA1c) measurement

Glycated hemoglobin A1c (HbA1c) was measured using a Cobas B101 POC system (Roche, Basel, Switzerland). Briefly, 2 μL heparinized blood was placed into the Cobas cartridge with TRIS buffer and sodium lauryl sulphate (SLS) buffer to form a SLS-Hb complex. Detection at 525 nm provided a measure of total Hb and the HbA1c fraction was measured via a latex agglutination immuno-turbimetric reaction at 625 nm. % HbA1c value was calculated using a ratio of the concentration of HbA1c to total Hb (n = 3/group).

### Histological analysis of collagen content

Formalin-fixed transverse mid-sections containing left and right ventricular myocardium were paraffin-embedded and sectioned (6 μm thickness) as previously described^57^. Sections were stained using picrosirius red (Picric Acid (Sigma Aldrich, MO, USA), 0.1% Sirius Red (BDH AnalaR, England)) for 60 minutes, dehydrated and mounted with DPX medium (Sigma-Aldrich, MO, USA). Brightfield microscopic images were captured using the Zeiss Imager D1 microscope connected to a Zeiss AxioCam MRc5 Colour Camera (Oberkochen, Germany) and AxioVision 40 acquisition software (version 4.7.1.0). Image analysis of 5 images per section, 1–2 sections per heart, 5 hearts per group, was performed in a blinded manner using Image Pro Plus (V4.5.1, Media Cybernetics, MD, USA). Images were subjected to grey scale (255 pixel range) transformation and a pixel intensity histogram was used to determine a non-biased threshold. A binary map of collagen deposition was generated, from which collagen density was calculated and expressed relative to the total number of pixels in the area of interest^57^.

### Gene expression analysis

RNA was extracted from frozen cardiac tissues (n = 3/group) using the TRIzol® reagent in conjunction with the PureLink™ Micro-to-Midi Total RNA Purification kit (Invitrogen, CA, USA) with on-column DNase treatment (PureLink DNase, Invitrogen, CA, USA). The RNA was reverse transcribed using the RT^2^ First Strand Kit (Qiagen, CA, USA) as per the manufacturer’s instructions. Real-time PCR was used to determine the relative gene expression levels using a customized RT^2^ PCR Array plate (Qiagen, CA, USA) on a Bio-Rad CFX thermocycler (Bio-Rad, CA, USA). The comparative ΔΔCt method was used to analyze the genes of interest relative to the β-actin reference gene as described^59^.

### Statistical analysis

Data are presented as mean ± SEM, with group samples sizes as indicated. Statistical analysis was performed using Graphpad Prism V7.0 (GraphPad, CA, USA). Data were analyzed by two-way ANOVA and Fisher’s LSD *post hoc* test performed to identify group differences when ANOVA significance was attained. A Student’s t-test was used for analysis of diabetic vs. control gene expression data for male and female separately (sex comparisons are not appropriate for these data). A p-value of <0.05 was considered statistically significant.

### Data availability

Data can be made available on request.
